# Supplementary material for: Superiority of Direct Oral Anticoagulants over Vitamin K Antagonists in Oncological Patients with Atrial Fibrillation: Analysis of Efficacy and Safety Outcomes
Source: J Clin Med. 2022 Sep 27;11(19):5712. doi: 10.3390/jcm11195712 (PMC9572823; doi:10.3390/jcm11195712)
Supplement: Supplementary file 1 [file jcm-11-05712-s001.zip › jcm-1836184-supplementary.pdf]

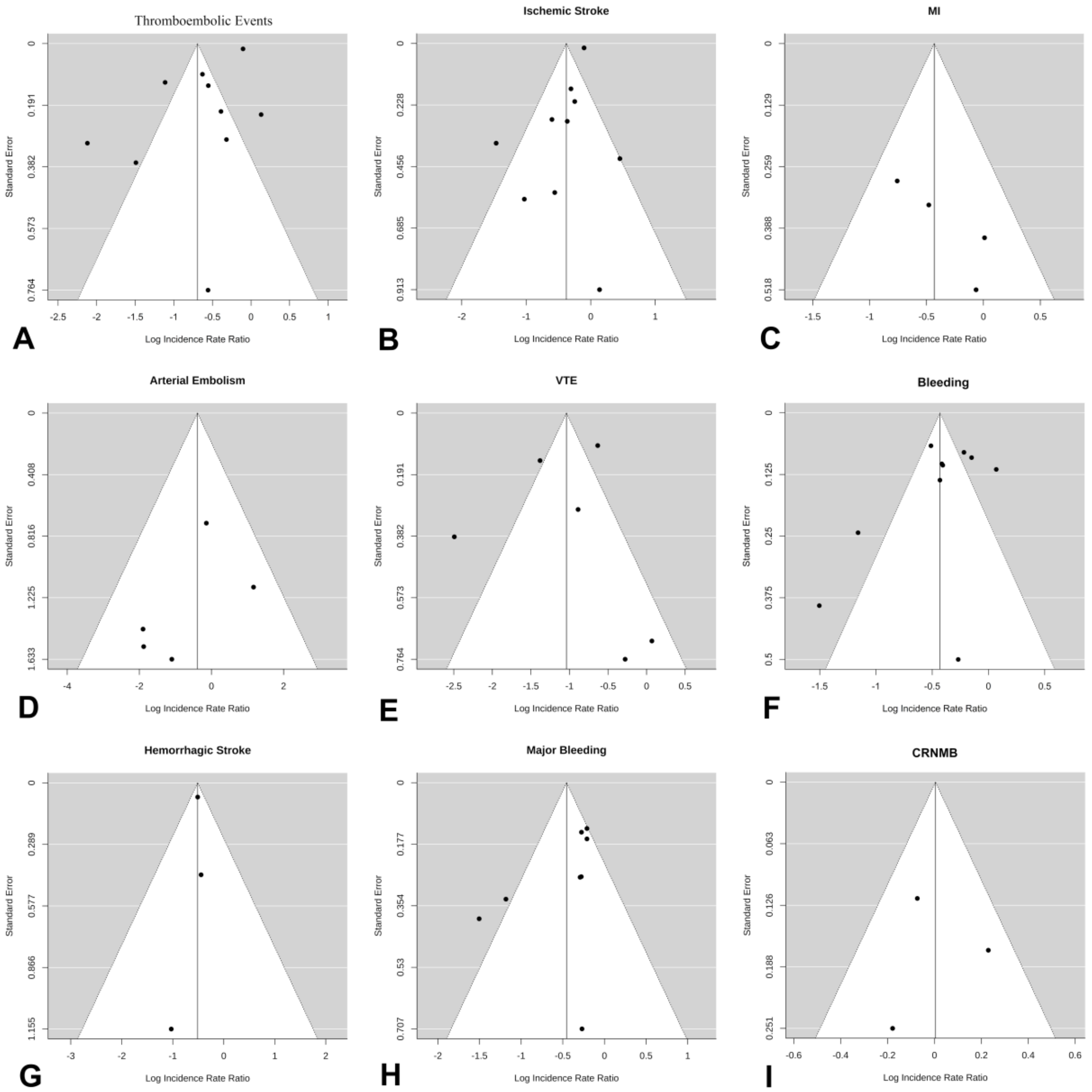

Figure S1. Funnel plots of efficacy and safety outcomes. **A.** Thromboembolic events (efficacy). **B.** Ischemic stroke. **C**. Myocardial infarction. **D.** Arterial embolism. **E.** Venous thromboembolic events. **F.** Any bleedings (safety). **G.** Hemorrhagic stroke. **H.** Major bleedings. **I.** Clinically relevant non-major bleeding.

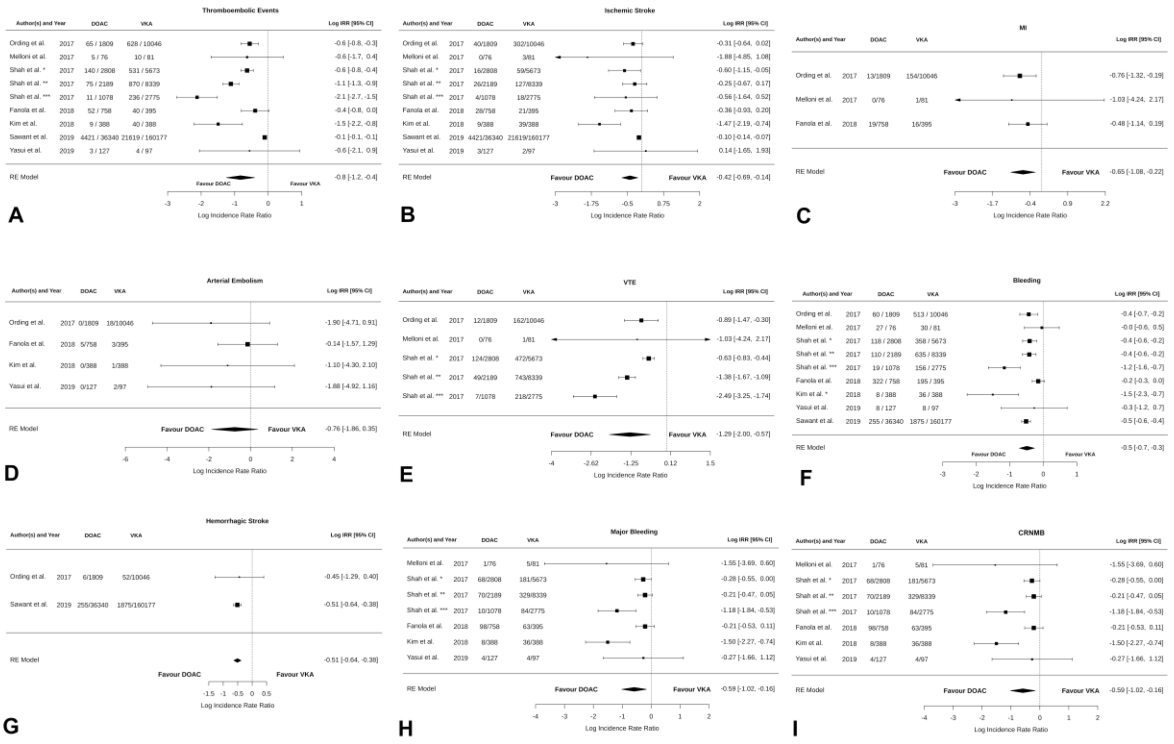

Figure S2. Forest plots of efficacy and safety outcomes in active cancer only patients. **A.** Thromboembolic events (efficacy). **B.** Ischemic stroke. **C**. Myocardial infarction. **D.** Arterial embolism. **E.** Venous thromboembolic events. **F.** Any bleedings (safety). **G.** Hemorrhagic stroke. **H.** Major bleedings. **I.** Clinically relevant non-major bleeding. *rivaroxaban, **dabigatran, ***apixaban

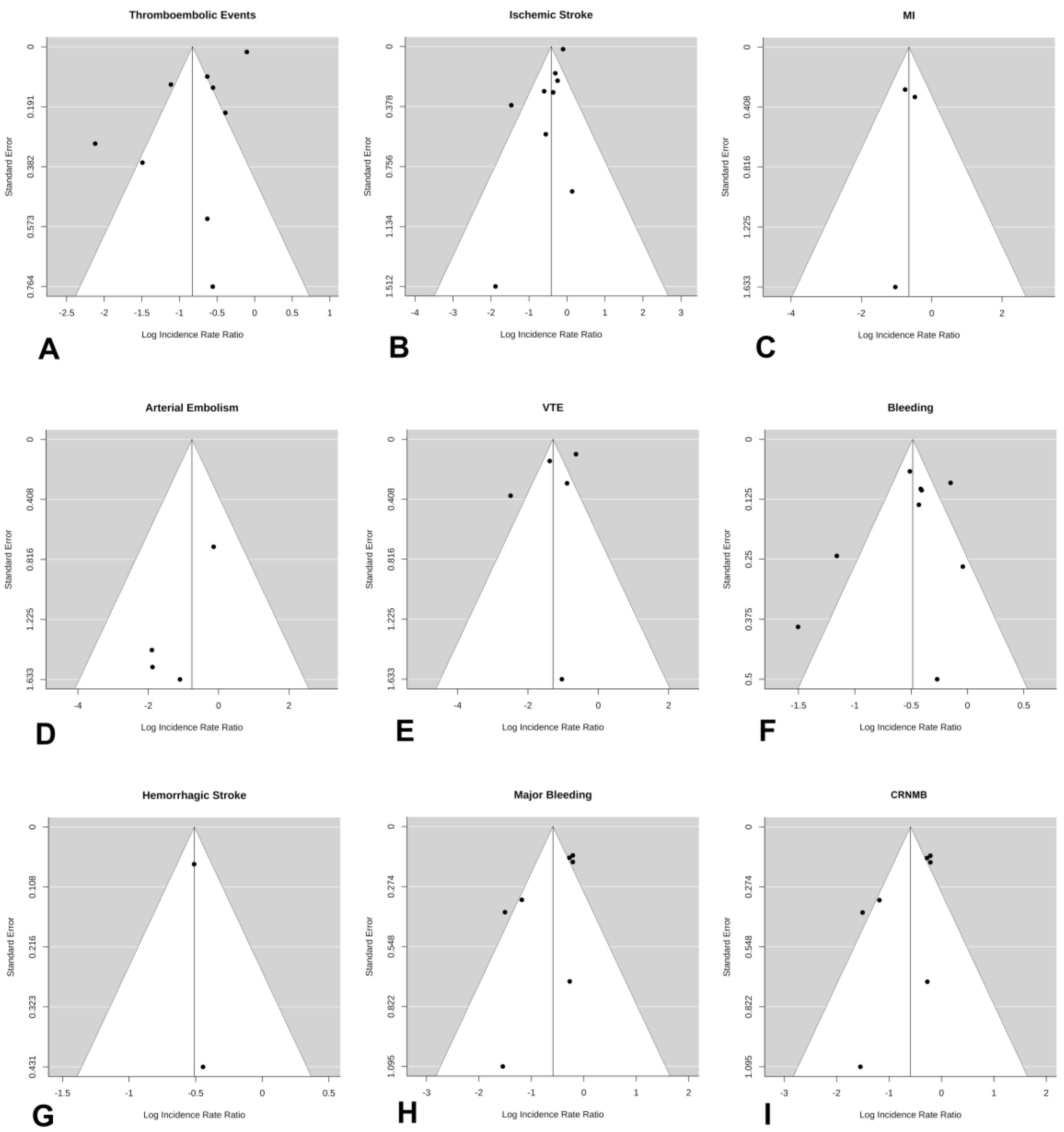

Figure S3. Funnel plots of efficacy and safety outcomes in active cancer only patients. **A.** Thromboembolic events (efficacy). **B.** Ischemic stroke. **C**. Myocardial infarction. **D.** Arterial embolism. **E.** Venous thromboembolic events. **F.** Any bleedings (safety). **G.** Hemorrhagic stroke. **H.** Major bleedings. **I.** Clinically relevant non-major bleeding.

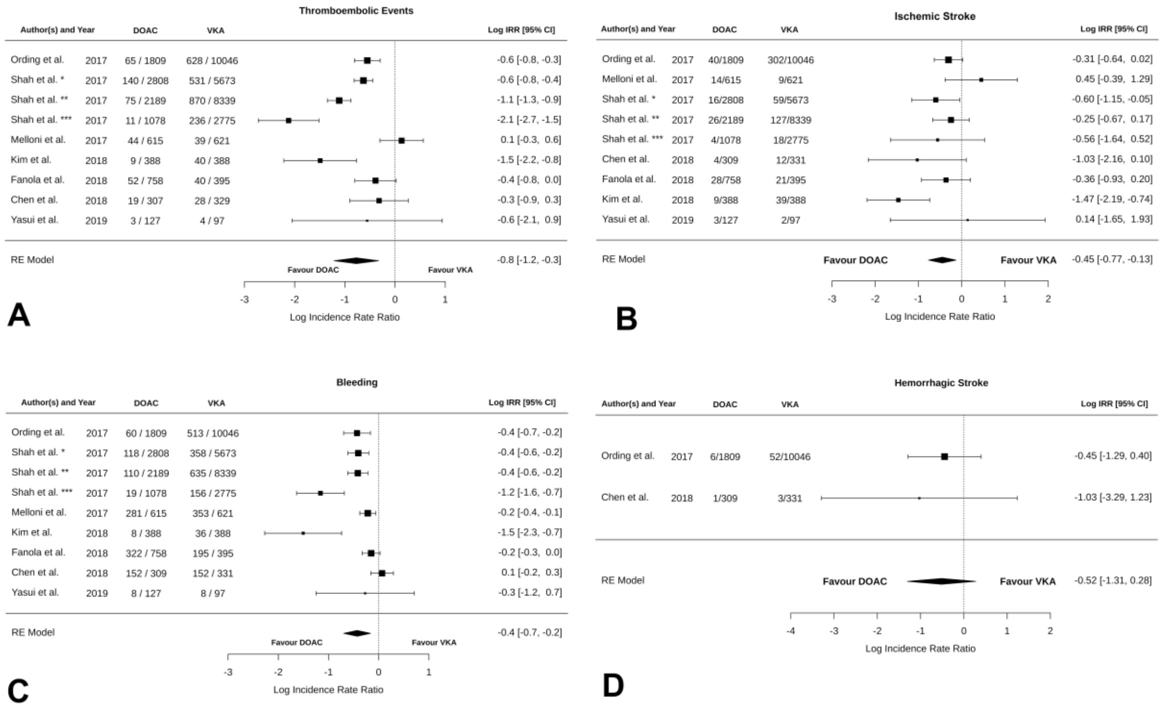

Figure S4. Forest plot of efficacy and safety outcomes without the study by Sawant et al. **A.** Thromboembolic events (efficacy). **B.** Ischemic stroke. **C.** Any bleedings (safety). **D.** Hemorrhagic stroke. *rivaroxaban, **dabigatran, ***apixaban

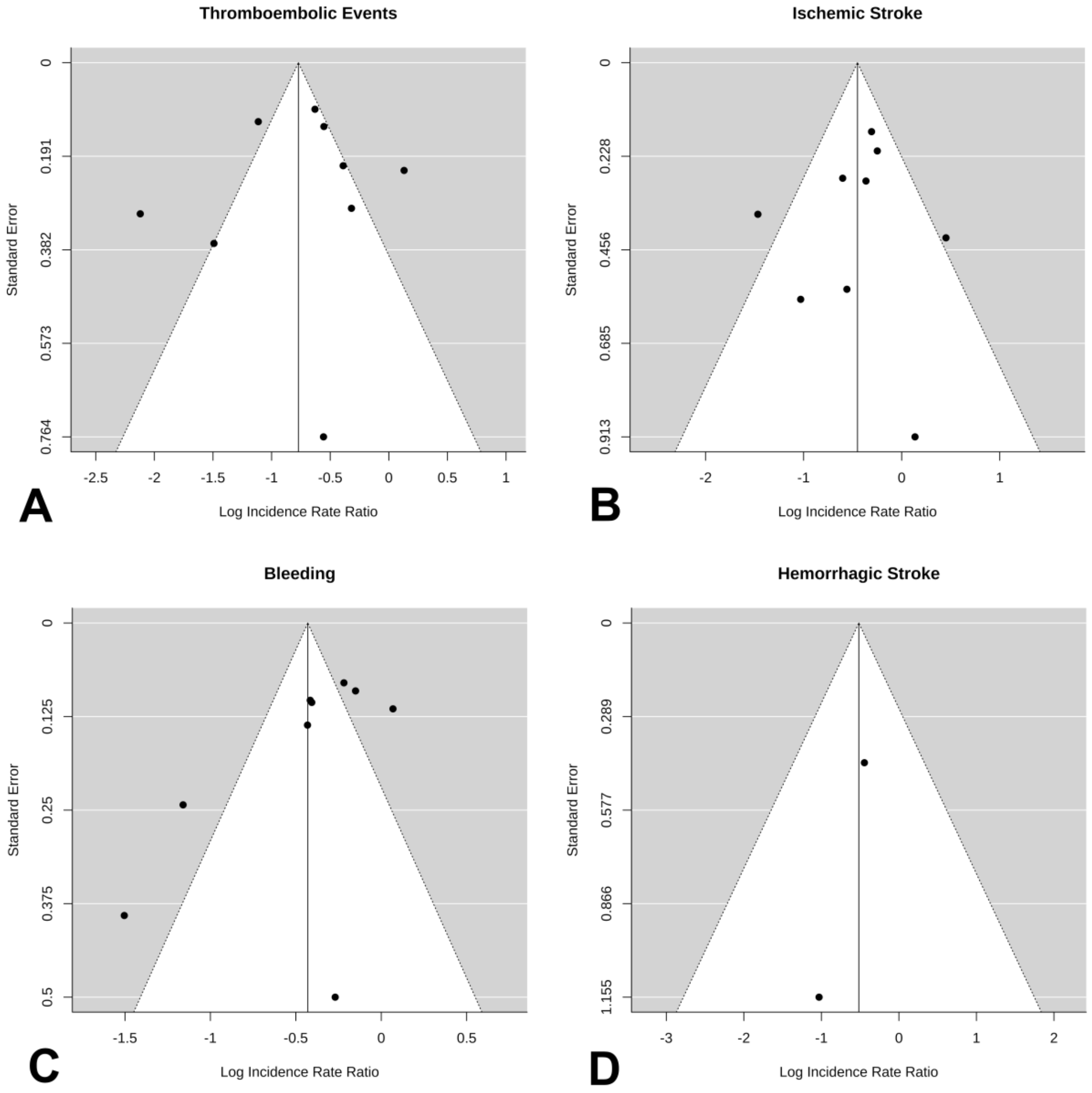

Figure S5. Funnel plot of efficacy and safety outcomes without the study by Sawant et al. **A.** Thromboembolic events (efficacy). **B.** Ischemic stroke. **C.** Any bleedings (safety). **D.** Hemorrhagic stroke.

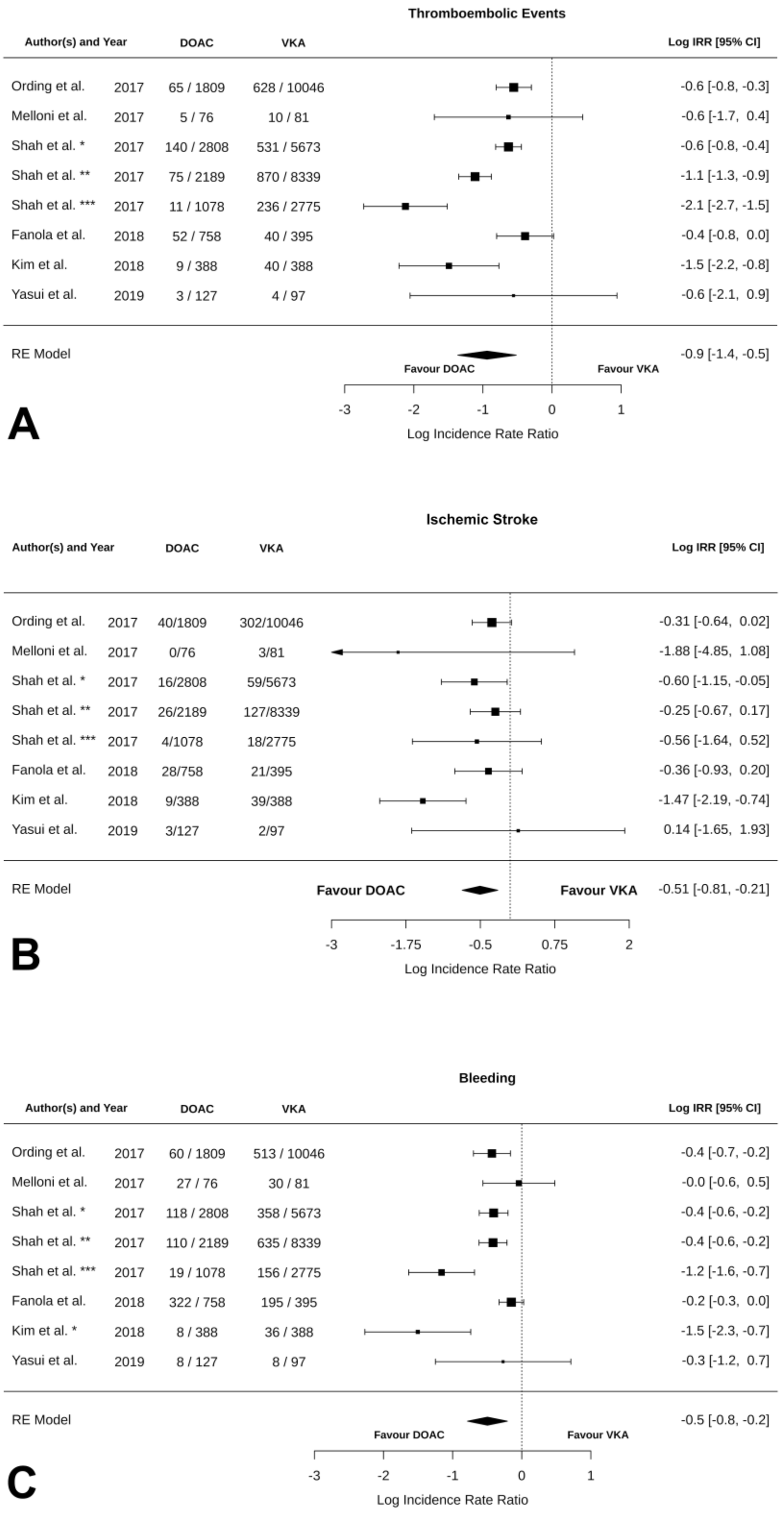

Figure S6. Forest plot of efficacy and safety outcomes without the study by Sawant et al. in active cancer only patients. **A.** Thromboembolic events (efficacy). **B.** Ischemic stroke. **C.** Any bleedings (safety). *rivaroxaban, **dabigatran, ***apixaban

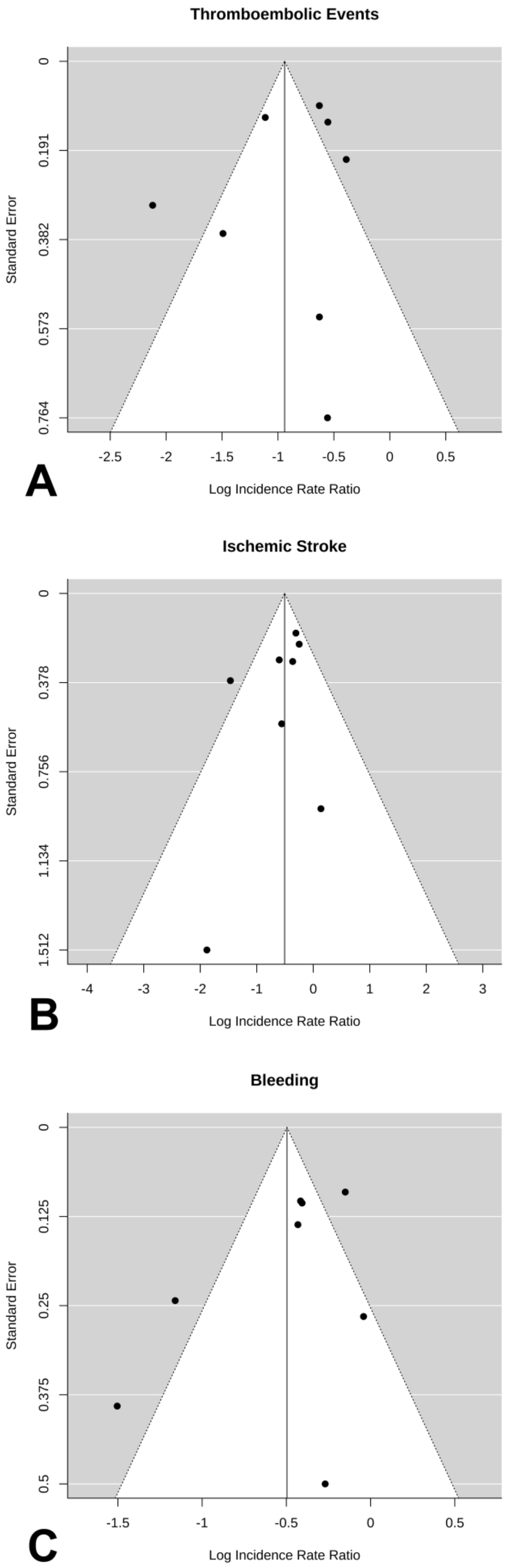
Thromboembolic Events
Standard Error
0
0.191
0.382
0.573
0.764
-2.5
-2
-1.5
-1
-0.5
0
0.5
Log Incidence Rate Ratio
A
Ischemic Stroke
Standard Error
0
0.378
0.756
1.134
1.512
-4
-3
-2
-1
0
1
2
3
Log Incidence Rate Ratio
B
Bleeding
Standard Error
0
0.125
0.25
0.375
0.5
-1.5
-1
-0.5
0
0.5
Log Incidence Rate Ratio
C

Figure S7. Funnel plot of efficacy and safety outcomes without the study by Sawant et al. in active cancer only patients. **A.** Thromboembolic events (efficacy). **B.** Ischemic stroke. **C.** Any bleedings (safety).

Supplemental Table S1. Dosages of DOACs and VKAs

Author	Year	VKA	DOAC
Ording et al.	2017	Not collected	Not collected
Shah et al.	2017	-	-
Melloni et al.	2017	Adjusted to INR	Apixaban: 5 mg twice daily (2.5 mg twice daily if: age >80 years, body weight <60 kg, serum creatinine >1.5 mg/dL)*
Kim et al.	2018	-	Dabigatran: 150 mg twice daily (110 mg twice daily if age ≥80 or eGFR = 30–50 mL/min)<br>Apixaban: 5 mg twice daily (2.5 mg twice daily if: age ≥80 or serum creatinine ≥1.5 mg/dL or body weight ≤60 kg)*<br>Rivaroxaban: 20 mg once daily (15 mg once daily if: eGFR = 15–49 mL/min)
Fanola et al.	2018	Adjusted to INR	Edoxaban: 30 mg or 60 mg once daily†
Chen et al.	2018	Adjusted to INR	Rivaroxaban: 20 mg once daily (15 mg once daily if creatinine clearance 30–49 mL/min)
Yasui et al.	2019	Not collected	Not collected
Sawant et al.	2019	-	-

The studies are shown in order of year of publication. Abbreviations: DOAC= Direct Oral Anticoagulants, eGFR= estimated Glomerular Filtration Rate, INR= International Normalized Ratio, VKA= Vitamin K Antagonists. *If 2 of these 3 criteria were present, †lower-dose and higher-dose, respectively.

Supplemental Table S2. Patient thrombotic and hemorrhagic risk: CHA_2DS_2 VASc score, CHA_2DS_2 score and HAS-BLED score

Author	Year	Overall			VKA group			DOAC group		
		CHA2DS2 VASc score	CHADS2 score	HAS-BLED score	CHA2DS2 VASc score	CHADS2 score	HAS-BLED score	CHA2DS2 VASc score	CHADS2 score	HAS-BLED score
Ording et al.	2017	-	-	-	-	-	-	-	-	-
Shah et al.	2017	-	-	-	4.6 ± 2.0	-	-	4.0 ± 2.0*<br>4.3 ± 2.0†<br>4.2 ± 1.9‡	-	-
Melloni et al.	2017	3.6 ± 1.52§<br>3.8 ± 1.42¶	2.2 ± 1.20§<br>2.3 ± 1.12¶	2.2 ± 0.99§<br>2.2 ± 1.02¶	-	-	-	-	-	-
Kim et al.	2018	-	-	-	3.6 ± 1.5	-	2.0 ± 1.1	3.6 ± 1.6	-	2.0 ± 1.0
Fanola et al.	2018	4.3 ± 1.4	2.8 ± 1.0	2.5 ± 1.0	-	-	-	-	-	-
Chen et al.	2018	-	3.5 ± 1.0	2.9 ± 0.9	-	-	-	-	-	-
Yasui et al.	2019	3.1 ± 1.4	1.9±1.2	2.0 ± 0.9	3.0 ± 1.5	1.9±1.1	2.1 ± 0.9	3.1 ± 1.4	2.0±1.3	2.0 ± 0.9
Sawant et al.	2019	-	-	-	-	-	-	-	-	-

The studies are shown in order of year of publication. Values are expressed as mean ± standard deviation. Abbreviations: VKA = Vitamin K Antagonists, DOAC = Direct Oral Anticoagulant.

* Rivaroxaban, † Dabigatran, ‡ Apixaban, § Active Cancer, ¶ Remote Cancer.

Supplemental Table S3. Malignancy characteristics

Author	Year	Type of cancer (Overall)							Type of cancer (VKA)							Type of cancer (DOAC)						
		GI	L	GU	H	B	IC	O	GI	L	GU	H	B	IC	O	GI	L	GU	H	B	IC	O
Ording et al.	2017	1,418 (12)	460 (3.9)	1,716 (14.5)	404 (3.4)	1,416 (11.9)	13 (0.1)	6,428 (54.2)	1,228 (12)	404 (4.0)	1,468 (15)	338 (3.4)	1,192 (12)	10 (0.1)	5,406 (54)	190 (11)	56 (3.1)	248 (14)	66 (3.6)	224 (12)	3 (0.2)	1,022 (56)
Shah et al.	2017	-	-	-	-	-	-	-	(13.9)	(13.1)	(28.7)	(10.1)	(17.8)	-	(16.4)	(10.3)* (10.7)† (11.6)‡	(12.5)* (10.4)† (8.6)‡	(29.0)*(31.3)† (29.8)‡	(9.6)* (9.2)† (8.8)‡	(21.4)* (20.8)† (23.4)‡	-	(14.6)* (15.4)† (15.8)‡
Melloni et al.	2017	200 (16.2)	37 (3)	555 (45)	36 (2.9)	199 (16.1)	-	206 (16.8)	-	-	-	-	-	-	-	-	-	-	-	-	-	-
Kim et al.	2018	-	-	-	-	-	-	-	138 (35.6)	44 (11.3)	94 (24.2)	9 (2.3)	8 (2.1)	13 (3.4)	82 (21.2)	138 (35.6)	51 (13.1)	69 (17.8)	8 (2.1)	11 (2.8)	19 (4.9)	92 (23.7)
Fanola et al.	2018	236 (20.5)	127 (11.0)	313 (27.1)	57 (4.9)	75 (6.5)	24 (2.1)	321 (27.8)§	-	-	-	-	-	-	-	-	-	-	-	-	-	-
Chen et al.	2018	122 (19.1)	20 (3.1)	303 (47.3)	33 (5.2)	94 (14.7)	27 (4.2)	102 (15.9)§	-	-	-	-	-	-	-	-	-	-	-	-	-	-
Yasui et al.	2019	99 (44.2)	54 (24.1)	25 (11.2)	7 (3.1)	9 (4.0)	22 (9.8)	8 (3.6)	45 (46.4)	27 (27.8)	8 (8.2)	1 (1.0)	2 (2.1)	10 (10.3)	4 (4.1)	54 (42.5)	27 (21.3)	17 (13.4)	6 (4.7)	7 (5.5)	12 (9.4)	6 (4.7)
want et al.	2019	-	-	-	-	-	-	-	-	-	-	-	-	-	-	-	-	-	-	-	-	-

The studies are shown in order of year of publication. Values are expressed as number (%). Abbreviations: B= Breast, DOAC = Direct Oral Anticoagulant, GI = Gastrointestinal, GU = Genitourinary, H = Hematological, IC = Intracranial, L = Lungs, O = Other, VKA = Vitamin K Antagonists. * Rivaroxaban, † Dabigatran, ‡ Apixaban, § other malignancies, multiple sites and unspecified malignancies.

Supplemental Table S4. Quality assessment

Item		M	SD
**1**	**Study hypothesis/aim/objective described?**	0.94	0.25
**2**	Main outcomes described in the introduction or methods?	1.00	0.00
**3**	Participant characteristics described?	1.00	0.00
**4**	Interventions of interest clearly described?	0.94	0.25
**5**	Distributions of principal confounders in each group of subjects to be compared clearly described?	1.50	0.52
**6**	Main findings described?	0.88	0.34
**7**	Estimates of the random variability in the data for the main outcomes provided?	1.00	0.00
**8**	All important adverse events reported?	1.00	0.00
**9**	Withdrawals and drop-outs reported?	0.53	0.52
**10**	Actual probability values been reported?	0.88	0.34
**11**	Contacted participants representative?	0.50	0.52
**12**	Prepared participants representative?	0.56	0.51
**13**	Staff, places, and facilities where the patients were treated, representative?	1.00	0.00
**14**	Were the subjects blinded the intervention they have received?	0.44	0.51
**15**	Was measuring the main outcomes of the intervention blinded?	0.38	0.50
**16**	Data dredging made clear?	1.00	0.00
**17**	Do the analyses adjust for different lengths of follow-up of patients?	0.94	0.25
**18**	Statistical tests appropriate?	1.00	0.00
**19**	Compliance with the intervention/s reliable?	0.94	0.25
**20**	Main outcome measures valid and reliable?	0.94	0.25
**21**	Participants recruited from the same population?	0.94	0.25
**22**	Participants recruited over the same time?	1.00	0.00
**23**	Subjects randomized to intervention groups?	0.44	0.51
**24**	Randomized intervention assignment complete and irrevocable?	0.38	0.50
**25**	Confounders controlled for?	0.44	0.51
**26**	Losses of patients to follow-up taken into account?	0.50	0.52
**27**	Sufficient power analysis provided?	1.56	2.39

All items have a maximum score of 1.00 except for item 5 and 27, which have a maximum score of 2.00 and 5.00, respectively.
